# Age Disparities in Frequency of Everyday Discrimination and Heightened Vigilance in a National United States Sample

**DOI:** 10.1093/geroni/igaf122.684

**Published:** 2025-12-31

**Authors:** Roger Wong, Karen Gonzalez, Kiersten Crawford

**Affiliations:** SUNY Upstate Medical University, Syracuse, New York, United States; SUNY Upstate Medical University, Syracuse, New York, United States; SUNY Upstate Medical University, Syracuse, New York, United States

## Abstract

Discrimination and vigilance are known to be crucial psychological factors that influence how individuals interact with others in their environment, however, whether these experiences vary across the lifespan remains poorly understood. Our present study compared age differences in the frequency of discrimination and vigilance. The 2023 National Health Interview Survey administered the validated five-item Everyday Discrimination Scale (e.g. treated with disrespect) and four-item Heightened Vigilance Scale (e.g. careful about your appearance) among a nationally representative sample of 28,583 adults in the United States. We categorized respondents into four age groups: young adult (18-24 years), early middle-age (25-39 years), late middle-age (40-64 years), and older adult (65+ years). Composite scores were created separately for discrimination (range 0-20) and vigilance (range 0-16) by adding all scale items. Average discrimination and vigilance scores were highest among young adults, but gradually decreased for each subsequent age group, which were statistically significant based on ANOVA tests. Two separate negative binomial regression models indicated compared to young adults, older adults had a significantly lower frequency of discrimination by 48% (Incidence Rate Ratio [IRR]=0.52, 95% CI = 0.48-0.57, p<.001) and vigilance by 39% (IRR=0.61, 95% CI = 0.56-0.66, p<.001), after applying complex survey sampling weights and adjusting for sociodemographic, geographic, and health covariates. These findings suggest that as age increases, frequencies of exposure to discrimination and exhibiting vigilant behaviors decrease. Further research is needed to explore the underlying mechanisms behind these age-related differences and how these associations may intersect with other sociodemographic factors such as race, gender, and sexual orientation.

